# Relationships of Trait Anxiety and Loss of Control Eating with Serum Leptin Concentrations among Youth

**DOI:** 10.3390/nu11092198

**Published:** 2019-09-12

**Authors:** Meghan E. Byrne, Marian Tanofsky-Kraff, Manuela Jaramillo, Lisa M. Shank, Sarah LeMay-Russell, Sarah G. Rubin, Sophie Ramirez, Deborah R. Altman, Natasha A. Schvey, Sheila M. Brady, Lauren B. Shomaker, Amber B. Courville, Shanna B. Yang, Merel Kozlosky, Miranda M. Broadney, Susan Z. Yanovski, Jack A. Yanovski

**Affiliations:** 1 Department of Medical and Clinical Psychology, Uniformed Services University of the Health Sciences (USUHS), 4301 Jones Bridge Road, Bethesda, MD 20814, USA; meghan.byrne@usuhs.edu (M.E.B.); mjaramillo480@gmail.com (M.J.); lisa.shank.ctr@usuhs.edu (L.M.S.); , natasha.schvey@usuhs.edu (N.A.S.); 2 Section on Growth and Obesity, Program in Endocrinology, Metabolism and Genetics, Division of Intramural Research, Eunice Kennedy Shriver National Institute of Child Health and Human Development (NICHD), National Institutes of Health (NIH), DHHS, 10 Center Drive, Bethesda, MD 20892, USA; sarah.rubin@nih.gov (S.G.R.); sophie.ramirez@nih.gov (S.R.); , bradys@mail.nih.gov (S.M.B.); miranda.broadney@nih.gov (M.M.B.); yanovskj@mail.nih.gov (J.A.Y.); 3 Metis Foundation, 300 Convent St #1330, San Antonio, TX 78205, USA; 4 Department of Human Development and Family Studies, College of Health and Human Sciences, Colorado State University, Fort Collins, CO 80523, USA; Lauren.Shomaker@colostate.edu; 5 Nutrition Department, Clinical Center, NIH, 10 Center Drive, Bethesda, MD 20892, USA; courvillea@cc.nih.gov (A.B.C.); shanna.bernstein@nih.gov (S.B.Y.); kozloskym@cc.nih.gov (M.K.); 6 Division of Digestive Diseases & Nutrition, National Institute of Diabetes, Digestive and Kidney Diseases (NIDDK), NIH, 6707 Democracy Blvd, Rm 6025, Bethesda, MD 20892, USA; yanovskis@extra.niddk.nih.gov

**Keywords:** loss of control eating, anxiety, leptin, dietary intake, child and adolescent, pediatric obesity

## Abstract

Loss of control (LOC) eating in youth is associated with elevated fasting serum leptin, even after accounting for adiposity. Anxiety is closely linked to, and may exacerbate, LOC eating. Yet, it remains unclear how anxiety relates to leptin, or if the relationship is moderated by the presence of LOC eating. We examined whether self-reported trait anxiety interacted with LOC eating in relation to leptin in a convenience sample of youths (*n* = 592; 13.1 ± 2.7 years; body mass index *z*-score (BMI*z*) = 0.9 ± 1.1; 61.8% girls; 53.5% non-Hispanic White; 36.6% with LOC eating). LOC eating was assessed by interview. Leptin was measured after an overnight fast. Exploratory analyses were conducted to examine anxiety and LOC eating in relation to laboratory intake patterns in three sub-samples. In a generalized linear model adjusting for relevant covariates, anxiety significantly interacted with LOC eating in relation to leptin (*p* = 0.02), such that greater trait anxiety related to higher concentrations of leptin only among youth with LOC eating. Trait anxiety was not significantly related to fasting serum leptin independently in a generalized linear model adjusting for age, race, height, sex, study type, and fat mass (kg). Exploratory mechanistic analyses of food intake patterns did not identify consistent results for participants with both anxiety and LOC eating. Among youth with LOC eating, anxiety may be associated with higher serum leptin. Prospective data are required to elucidate the directionality and mechanisms of these relationships.

## 1. Introduction

Pediatric loss of control (LOC) eating is associated with obesity [1] and has been shown to predict excess weight gain [2,3,4]. LOC eating involves the subjective experience of feeling out of control while eating and is a hallmark feature of binge-eating disorder. Unlike classic binge episodes that require the consumption of an unambiguously large amount of food, LOC eating is considered present whenever the experience of feeling out of control is reported, even when the amount of food consumed is not clearly large. Few children meet full syndrome criteria for binge-eating disorder [1,5], but about 31% of children and adolescents with overweight and obesity report experiencing at least occasional episodes of LOC eating [6]. Due to the maturation of brain and cognitive functioning, as well as relationships with peers gaining a more central role [7], adolescence is considered a particularly critical time for the development of disordered eating behaviors including LOC eating [8]. Although the exact mechanisms relating LOC eating to excess body weight are not entirely understood, the behavior has been cross-sectionally and prospectively associated with adverse metabolic outcomes among youth, independent of adiposity [9,10,11].

Among the numerous adverse health-related consequences related to LOC eating is dysregulated serum leptin. Serum leptin is an adipose-tissue-derived hormone that modulates hunger and food intake through signaling satiety, and is essential for normal body weight regulation [12,13]. Compared to their counterparts without LOC, youth with LOC eating have higher concentrations of fasting serum leptin, even after adjusting for adiposity [14]. Leptin is highly associated with body fat mass [15] and has been shown to increase acutely in response to positive energy balance [16]. High leptin may be an appropriate piece of the body’s counter-regulatory response attempting to reverse adverse outcomes as a result of excess energy intake. Yet, high serum leptin may also indicate leptin resistance, in that the central nervous system develops a resistance to leptin’s regulatory actions, which could mechanistically lead to maintenance or exacerbation of overeating [17]. Therefore, the relation between serum leptin and LOC eating warrants further investigation.

Despite the adverse outcomes related to pediatric LOC eating, not all youth who report LOC eating have persistent LOC, with roughly half of youth with LOC eating reporting remission over time in the absence of intervention [18,19,20]. This suggests the need to identify potential risk or maintenance factors that can help to distinguish individuals with LOC eating who will prospectively develop problematic behavioral and metabolic outcomes from those who will not. One such factor may be anxiety. Anxiety has been consistently linked to LOC eating in youth [21,22], and LOC eating has been shown to predict increases in anxiety symptoms over time [18]. Although a number of negative affective factors have been linked to LOC eating in the literature, anxiety has been found to be even more salient than other negative mood states, including depression, confusion, fatigue, and anger, in relation to LOC eating behaviors among youth [23]. Notably, links between anxiety and LOC eating have been found above and beyond the contribution of excess body weight or fat [22]. In the absence of LOC eating, anxiety has been associated with obesity in pediatric samples [24]. Moreover, in youth with, but not without, LOC eating, trait anxiety was found to be significantly related to fasting insulin and insulin resistance cross-sectionally, even after adjusting for differences in adiposity [9].

Conjointly, both insulin and leptin have been implicated as regulators of the rewarding value of food [17], suggesting that these hormones are important to consider in models of LOC eating and risk for excess weight gain. Neural regions implicated in aspects of the rewarding and motivating value of food are known to be activated during times of stress [25]. Release of leptin has been shown to be similarly activated as a part of the stress response [26,27]. Further, leptin has been implicated as a biomarker of stress perception in a college sample of females aged 19–21 years [28]. However, anxiety, a prominent facet of psychosocial stress, has not been well-studied in relation to serum leptin among youth with LOC eating. It is unclear whether anxiety is associated with increased serum leptin in the absence of LOC eating, or whether LOC eating is necessary for such a relation to emerge. Taken together, examining anxiety in relation to serum leptin may help clarify those youth with LOC eating who are potentially at the highest risk for persistent LOC and adverse outcomes.

Therefore, we studied whether trait anxiety is related to serum leptin in children and adolescents. We further examined whether the relationship between trait anxiety and fasting serum leptin was moderated by LOC eating. We hypothesized that trait anxiety would be positively related to fasting serum leptin, and that anxiety would interact with LOC eating, such that among those with LOC eating, anxiety would be more robustly associated with serum leptin. Given data suggesting that fat macronutrient intake may mediate the relationship between leptin and binge eating [29], and that LOC eating is associated with more carbohydrate and less protein consumption [30], we also explored overall caloric intake and macronutrient intake patterns in laboratory test meals to examine potential mechanisms for the relationship between anxiety, LOC eating, and leptin.

## 2. Materials and Methods

### 2.1. Participants

A convenience sample of youths, ranging primarily from middle childhood to adolescence (ranging 8–18 years; *m* = 13.1 years), was aggregated from three separate studies, one of which was jointly conducted by the Intramural Research Program of the *Eunice Kennedy Shriver* National Institute of Child Health and Human Development (NICHD), National Institutes of Health (NIH) and the Uniformed Services University of the Health Sciences (USUHS), and two of which were conducted by NICHD. Study 1 (ClinicalTrials.gov ID: NCT00680979, 08-CH-0139) consisted entirely of intervention-seeking adolescent girls with a body mass index (BMI, kg/m^2^) between the 75th and 97th percentiles who reported at least one episode of LOC eating in the past month. All participants in Study 1 were studied at a baseline screening visit prior to receiving any intervention. Studies 2 and 3 (ClinicalTrials.gov IDs: NCT00320177, 04-CH-0050; NCT02390765, 15-CH-0096) consisted of healthy, non-intervention-seeking boys and girls across the weight spectrum. The latter studies were designed to examine eating behaviors that promote pediatric overweight and obesity and associated health comorbidities.

Participants were recruited through physicians’ offices, local newspaper advertisements, and mailings to families in the greater Washington, DC metropolitan area. All participants were English-speaking. Those with a major medical or obesity-related illness, use of medications known to affect weight, recent weight loss, pregnancy, presence of a major psychological disorder, or a history of an eating disorder other than binge-eating disorder were excluded from enrollment. These protocols were approved by the institutional review boards of NICHD and USUHS. Written informed consent from parents and assent from youth were obtained.

### 2.2. Procedures

Participants were studied at the NIH Hatfield Clinical Research Center. All participants completed physiological assessments following an outpatient overnight fast starting at 10:00 p.m. the night before their laboratory visit. Questionnaires and semi-structured interviews were completed at a separate screening visit.

### 2.3. Measures

#### 2.3.1. Body Composition 

Height was measured in triplicate by a stadiometer. Weight was measured by a scale calibrated to the nearest 0.1 kg. BMI (kg/m^2^) was calculated for all participants using average height across three measurements and weight. Age and sex were used in calculations of BMI standard deviation scores (BMI*z*) according to the Centers of Disease Control and Prevention growth standards [31]. Total mass (kg), lean mass/fat-free mass (kg), fat mass (kg), and fat mass percentage (%) were assessed by either dual-energy X-ray absorptiometry (DXA) or air displacement plethysmography (Bod Pod), depending on the study protocol. To achieve near-equivalence between the two assessment techniques for percent adiposity, measurements of adiposity from the Bod Pod were adjusted by multiplying girls’ Bod Pod fat mass percentage by 1.03 [32].

#### 2.3.2. Serum Leptin 

Fasting blood samples were collected by a trained phlebotomist or registered nurse. Serum leptin concentrations were determined by commercially available immunoassays (Linco, St. Louis, MO or Mayo medical laboratories, New England, USA).

#### 2.3.3. State-Trait Anxiety Inventory for Children-Trait Scale 

Youth completed the State-Trait Anxiety Inventory for Children (STAIC) trait scale [33], a 20-item self-report measure of trait anxiety. Trait anxiety is defined as a personality trait capturing individual differences in the likelihood of experiencing anxiety across different situations [34]. The trait anxiety subscale includes items such as “I worry too much,” rated as “hardly ever,” “sometimes,” or “often.” Subscale scores range from 20 to 60. The STAIC does not identify a clinical cutoff score, however the measure has demonstrated good reliability and construct validity [35]. In the current sample, Cronbach’s α was 0.90.

#### 2.3.4. Eating Disorder Examination 

The Eating Disorder Examination (EDE) [36] or the EDE adapted for children [37], was administered to determine whether participants endorsed LOC eating. The original and child versions of the EDE have been effectively combined in previous studies [38]. Participants who endorsed at least one LOC eating episode within the past month were coded as “LOC present.” Additionally, behavioral and cognitive dietary restraint is captured in the Restraint subscale of the EDE, which was considered as a covariate in analyses for serum leptin, given the established relationship between restraint and serum leptin [29]. The EDE has demonstrated good to excellent test-retest and interrater reliability in youth across the weight spectrum [21,39]. The child EDE has demonstrated good to excellent interrater reliability [5,40], and good internal consistency and discriminant validity among youth [40].

#### 2.3.5. Laboratory Test Meals for Exploratory Mechanism Analyses 

Exploratory analyses of total energy intake (kcal) and percentage of energy from macronutrients were conducted using data from laboratory buffet test meals comprised of an array of foods varying in macronutrients. Intervention-seeking girls in Study 1 (ClinicalTrials.gov ID: NCT00680979, 08-CH-0139) participated in a ~9385-kcal array buffet lunch paradigm, consisting of 51% carbohydrate, 12% protein, and 37% fat, at 11:00 a.m. following an overnight fast. The buffet lunch test meal was consumed at a baseline screening visit prior to participation in an intervention for prevention of excessive weight gain. Non-intervention-seeking youth in Study 2 (ClinicalTrials.gov ID: NCT00320177, 04-CH-0050) consumed the same lunch array at 2:30 p.m. after consuming a standardized breakfast (i.e., 240 mL apple juice, 1 English muffin, 6 g butter) at 8:40 a.m. Participants in Study 3 (ClinicalTrials.gov ID: NCT02390765, 15-CH-0096) consumed lunch at 12:30 p.m. from a >10,000 kcal array consisting of 54% carbohydrate, 12% protein, and 33% fat, after consuming a body-weight standardized breakfast shake (consisting of 17% protein, 16% fat, and 67% carbohydrate) at 10:00 a.m. Participants across all protocols received standardized tape-recorded instructions to “Let yourself go and eat as much as you want”, immediately prior to the test meal. This LOC eating test meal instruction is well-validated and has been successfully used in pediatric [30] and adult studies [41]. Macronutrient content for each food item was determined according to standards from the U.S.D.A. Nutrient Database for Standard Reference and package information from Nutrition Facts Labels. Consumption (total kcal) was calculated by weighing each item before and after the meal. Percentage macronutrient intake was calculated by dividing the number of calories consumed from a given macronutrient by the number of total calories consumed.

### 2.4. Statistical Analyses

All statistical analyses were conducted using IBM SPSS Statistics for Macintosh, Version 25.0 (IBM Corp., Armonk, NY, USA). Data were screened for outliers, skew, and kurtosis. Influential outliers (<1% of data points) were recoded to fall within 1.5 times the interquartile range above or below the 25th or 75th percentile, respectively [39]. Fasting serum leptin did not achieve normality and was thus logarithmically-transformed (base 10) for analysis.

A univariate generalized linear model was utilized to analyze associations of trait anxiety, LOC eating, and the interaction of anxiety and LOC with serum leptin, adjusting for age, race, height (cm), sex, study type (i.e., intervention-seeking versus non-intervention-seeking), and fat mass (kg). This analysis was conducted in the total aggregate sample as well as in each study sample separately. A secondary analysis controlling for adiposity by including BMI*z* instead of fat mass found similar relationships and thus is not separately reported. Given the relationship between dietary restraint and leptin [26], EDE restraint (dichotomized into “presence versus absence” due to skewed distribution of data) was considered as a covariate in the model for leptin, however, it did not significantly contribute and was removed from analyses.

In exploratory follow-up analyses, we analyzed anxiety in relation to total energy intake and percent of energy consumed from macronutrients to provide a potential mechanism for the pattern of findings. Given the differences in laboratory test meal composition and timing, analysis models were conducted for each of the three study meals separately. To correct for multiple comparisons, multivariate generalized linear models of total intake (kcal) and macronutrient percentage intake from “sweet fats” (i.e., the summative combination of carbohydrate and fat consumed) and protein were analyzed in the three separate samples. Arcsine transformations were conducted for percentage intake from sweet fats and protein.

The first exploratory analysis consisted of intervention-seeking girls with a BMI between the 75th–97th percentile who reported at least one episode of LOC eating in the past month (Study 1). Thus, models predicting percent of energy consumed from macronutrients in intervention-seeking girls (Study 1) adjusted for age, race, height (cm), lean mass (kg), and body fat mass (%), with anxiety as the independent variable. In the second and third exploratory analyses, the models predicting percent of energy consumed from macronutrients for the studies of non-intervention-seeking youth (Studies 2 and 3, conducted separately) adjusted for age, race, height (cm), sex, lean/fat-free mass (kg), and body fat mass (%), with anxiety, LOC eating, and the interaction of anxiety by LOC eating as the independent variables. Models of percent of energy consumed from macronutrients were also analyzed without body composition covariates (i.e., lean/fat-free mass and body fat mass) included, but did not significantly differ from models including body composition and thus the covariates were retained.

## 3. Results

### 3.1. Participant Characteristics

The aggregated sample was comprised of 592 youth (13.1 ± 2.7 years) and was 61.8% female. Over one-third (36.6%) of the sample endorsed LOC eating. Participants were 53.5% non-Hispanic White, 29.1% Black or African American, 6.3% Asian, and 7.8% Hispanic/Latino. Average BMI*z* was 0.9 ± 1.1, ranging from −2.4 to 3.3, and average BMI percentile was 72.8 ± 27.6, ranging from 1.0 to 99.9. Table 1 outlines participant characteristics for the total sample and compares mean differences on primary variables by each study. Consistent with prior literature [20], a one-way ANOVA showed that trait anxiety was significantly higher in youth who reported LOC eating compared to youth without LOC (*F*(1, 560) = 71.58, *p* < 0.01).

### 3.2. Serum Leptin

In a generalized linear model adjusting for age, race, height, sex, study type, and fat mass (kg), trait anxiety was not significantly related to fasting serum leptin (*F*(1, 511) = 3.00, *p* = 0.08) in the total aggregate sample. Consistent with prior findings [14], the main effect of LOC eating was significantly related to concentrations of fasting serum leptin (*F*(1, 511) = 5.81, *p* = 0.02) in the total sample (Table 2).

**Figure 1:**
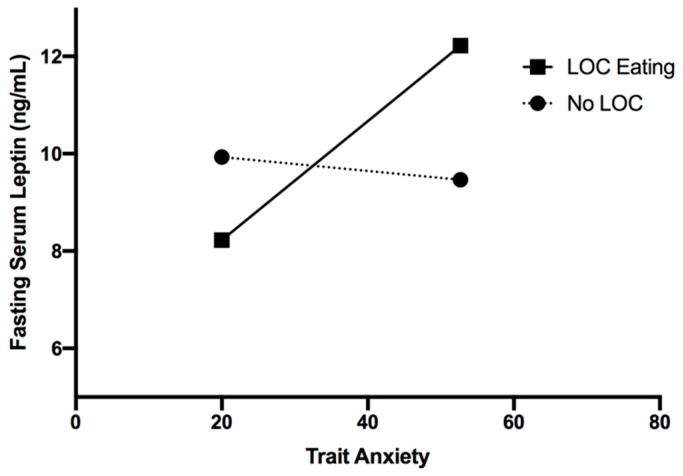
Interaction of LOC eating and trait anxiety in relation to fasting serum leptin (*p* = 0.02).

The interaction of anxiety and LOC eating, however, was significantly related to fasting serum leptin (*F*(1, 511) = 5.27, *p* = 0.02) in the total sample (Table 2), such that only among youth with LOC eating, higher trait anxiety was related to higher concentrations of fasting serum leptin (Figure 1).

Analyses for leptin were also conducted in each study sample separately. Given the fact that all girls in Study 1 endorsed LOC eating, the main effect of trait anxiety was examined on fasting serum leptin, adjusting for age, race, height (cm), and fat mass (kg). Trait anxiety was significantly related to fasting serum leptin (*F*(1, 104) = 6.08, *p* = 0.02) in Study 1 girls with LOC eating. In Study 2, the interaction of anxiety and LOC eating on fasting serum leptin was significant (*F*(1, 207) = 4.10, *p* = 0.04), adjusting for age, race, height, sex, and fat mass (kg). In Study 3, the interaction of anxiety and LOC eating was not significantly related to fasting serum leptin (*F*(1, 200) = 0.54, *p* = 0.47), after adjusting for the same covariates as Study 2.

### 3.3. Exploratory Follow-Up Analyses

#### 3.3.1. Sample 1, Intervention-Seeking Girls with Loss of Control (LOC) Eating

Given that all girls endorsed LOC eating in Study 1, main effects of trait anxiety were examined, adjusting for age, race, height (cm), lean mass (kg), and body fat mass (%). Results showed no significant relationship between the main effect of anxiety and overall intake (kcal) (*F*(1, 108) = 2.57, *p* = 0.11). Among girls in Study 1, there was a significant positive relationship between trait anxiety and percentage of energy consumed from sweet fats (*F*(1, 108) = 5.16, *p* = 0.03), and conversely a significant negative relationship between trait anxiety and percentage of energy consumed from protein (*F*(1, 108) = 5.11, *p* = 0.03).

#### 3.3.2. Sample 2, Non-Intervention-Seeking Boys and Girls

The main effects of trait anxiety in Study 2 were examined in addition to the interaction of anxiety by LOC eating on total energy intake and percentage of energy from macronutrients, adjusting for age, race, height (cm), sex, fat-free mass (kg), and body fat mass (%). There was no pattern of significant relationships between the main effect of trait anxiety and intake patterns, including total energy intake (kcal) (*F*(1, 195) = 0.05, *p* = 0.82), percentage of energy consumed from sweet fats (*F*(1, 195) = 0.08, *p* = 0.78), or protein (*F*(1, 195) = 0.14, *p* = 0.71). The interaction of trait anxiety by LOC eating on macronutrient intake patterns was similarly not significant for total energy intake (kcal) (*F*(1, 195) = 3.20, *p* = 0.08), percentage of energy consumed from sweet fats (*F*(1, 195) = 0.26, *p* = 0.61), or percentage of energy consumed from protein (*F*(1, 195) = 0.58, *p* = 0.45). Main effects of trait anxiety were also analyzed in models without LOC eating or the interaction term of LOC eating by anxiety included, and there were similarly no significant relationships between anxiety and total energy intake (*p* = 0.38), percentage of energy consumed from sweet fats (*p* = 0.59), or percentage of energy consumed from protein (*p* = 0.60).

#### 3.3.3. Sample 3, Non-Intervention-Seeking Boys and Girls

The main effects of trait anxiety in Study 3 were examined in addition to the interaction of anxiety by LOC eating on total energy intake and percentage of energy consumed from macronutrients, adjusting for age, race, height (cm), sex, lean mass (kg), and body fat mass (%). There was no pattern of significant relationships between the main effect of trait anxiety and dietary intake patterns, including total energy intake (kcal) (*F*(1, 193) < 0.01, *p* = 0.99), percentage of energy consumed from sweet fats (*F*(1, 193) = 0.08, *p* = 0.78), or protein (*F*(1, 193) = 0.05, *p* = 0.83). The interaction of trait anxiety by LOC eating on macronutrient intake patterns was similarly not significant for total energy intake (kcal) (*F*(1, 193) = 0.73, *p* = 0.40), percentage of energy consumed from sweet fats (*F*(1, 193) = 0.03, *p* = 0.87), or percentage of energy consumed from protein (*F*(1, 193) = 0.03, *p* = 0.86). Similar to Study 2, main effects of trait anxiety were also analyzed in models without LOC eating or the interaction term of LOC eating by anxiety, and there were no significant relationships between anxiety and total energy intake (*p* = 0.52), percentage of energy consumed from sweet fats (*p* = 0.47), or percentage of energy consumed from protein (*p* = 0.54).

## 4. Discussion

In a large sample of youth of a broad age and weight range, the interaction between anxiety and LOC eating was significantly related to fasting serum leptin, such that only among youth with LOC eating, higher trait anxiety was related to higher concentrations of fasting serum leptin. Trait anxiety was not significantly related to fasting serum leptin independently in a generalized linear model adjusting for age, race, height, sex, study type, and fat mass (kg). Exploratory analyses did not show a consistent relationship between anxiety or the interaction of anxiety with LOC eating on total intake or percentage of energy consumed from sweet fats or protein among youth in the non-intervention-seeking samples. However, there was a significant association between trait anxiety and higher percentage energy consumed from sweet fats and lower percentage of energy consumed from protein in the intervention-seeking sample of girls, all of whom reported LOC eating.

Elevated leptin out of proportion to fat mass may be an indicator of leptin resistance. It is possible that those with both anxiety symptoms and LOC eating are at particular risk for leptin resistance and excess weight gain [14,42]. Affect theory, a commonly supported model in the eating disorders literature, may help to explain the connection between anxiety and LOC eating as it proposes that negative affect leads to LOC eating as a maladaptive method to cope with negative emotions [43,44,45]. Research suggests that food intake activates neurological reward pathways that reduce negative affective states [46], such as anxiety. Elevated levels of serum leptin can impair dopaminergic functioning [17] and thus youth with LOC eating and elevated leptin may be driven to consume increased amounts of palatable foods in response to anxiety as a maladaptive attempt to cope with negative emotions. It is possible that anxiety may relate to greater intake of palatable foods and lower consumption of energy from protein among a high-risk subgroup of intervention-seeking girls with LOC eating who already meet criteria for overweight or obesity. Alternately, examining food intake outside of the laboratory in a naturalistic setting may be an important direction of study to better understand more ecologically valid eating patterns among youth. Examination of cross-sectional data, as in the current study, is the first step in supporting these potential mechanisms.

There may be other mechanisms that could explain the relationships among anxiety, LOC eating, and serum leptin that were not captured in the current analyses. Higher cortisol, a physiological marker of anxiety/stress, has been associated with higher leptin concentrations in girls [47]. Elevated cortisol can stimulate appetite through various biological pathways, including decreasing neural sensitivity to leptin and contributing to leptin resistance [48]. Some research [49,50] has demonstrated that adults with binge-eating disorder have higher cortisol following stressors compared to healthy controls. One possibility is that a combination of high cortisol, potentially due to higher trait anxiety, and elevated leptin may drive youth with LOC eating to engage in “stress eating,” and thus increase the risk for excess weight gain and the development of adverse obesity-related health markers. Cortisol was not examined in the current study and may be an important factor to consider in future research. Further prospective and mechanistic data are needed to clarify the nature of the relationship among leptin and LOC eating, and whether anxiety may drive this link.

Strengths of the current study include a large sample size and inclusion of a racially diverse sample of boys and girls, improving the generalizability of the current findings. Well-validated semi-structured interviews were used to assess LOC eating. We also collected objective measures of body composition and serum leptin. Limitations include the use of a self-report questionnaire to assess trait anxiety, as well as use of cross-sectional data, restricting the ability to draw causal relationships between trait anxiety and dysregulated leptin. Given discrepancies in the methodology between different studies’ test meals, the current analyses were limited in the ability to examine intake of certain types of food among the total sample. It is possible that the intake of certain palatable foods may be differentially influenced by the combination of anxiety and LOC eating in youth. Additionally, discrepancies in test meal methodology between studies resulted in differences in fasting versus non-fasting states during other data collection procedures, which may pose a limitation. However, this does not apply to trait anxiety, the primary independent variable of interest, as this trait measure questionnaire was collected when participants were not fasted in each of the studies and is considered to capture stable qualities over time, as opposed to state anxiety. Leptin values were collected in a standardized fashion in the morning, and thus would likely not be affected study-related differences.

This study evaluated a possible mechanism related to LOC eating, and may distinguish youth at potentially increased risk of excess weight gain. One mechanism for excess weight gain may be exacerbated LOC eating in response to underlying trait negative affect, with energy intake further driven by leptin resistance, irrespective of body fat. If supported by longitudinal data, these findings could contribute to our understanding of risk for obesity in youth, possibly identifying youth with LOC eating and greater trait anxiety as a high-risk group for weight gain potentially through dysregulated leptin. Future research should continue to clarify mechanisms, such as increased release of cortisol or increased consumption of certain types of food, that may uniquely influence the risk for excess weight gain among youth who endorse greater trait anxiety and LOC eating. Additionally, routine screening for trait anxiety and targeted use of intervention techniques to lower anxiety symptoms in youth with LOC eating should be studied for their ability to reduce LOC eating and in turn, reduce the clinical burden of excessive weight gain and adverse outcomes. Clinicians should also take into consideration other indirect factors that are strongly associated with both leptin and anxiety, such as adiposity, when conducting and interpreting any screening measures to inform individualized treatment approaches for youth at risk of excess weight gain.

## 5. Conclusions

Among youth with LOC eating, anxiety may be related to greater dysregulation of serum leptin. Increases in leptin may be a counter-regulatory response attempting to reverse excess weight gain induced by exacerbated LOC eating. However, more research is needed to clarify the directionality of these relationships prospectively and to better understand how anxiety relates to serum leptin in youth with LOC eating. Given that exploratory analyses of intake patterns were inconclusive as a potential mechanism for this relationship, future research is warranted. Research should continue to examine potential mechanisms prospectively in order to better inform targeted interventions focused on youth at the greatest risk for excess weight gain and adverse obesity-related outcomes.

## Figures and Tables

**Table 1:** Sample characteristics for total sample and by study.

	Total Sample (*n* = 592)	Study 1 (*n* = 113)	Study 2 (*n* = 239)	Study 3 (*n* = 240)
Trait Anxiety	31.4 ± 7.1	34.1 ± 6.8 *	30.5 ± 6.6	31.0 ± 7.4
LOC ^a^ presence (%)	36.6	100 *	29.4	12.3 *
Female (%)	61.8	100 *	50.6	55.0
Non-Hispanic White (%)	53.5	60.2	55.6	48.3
Black or African American (%)	29.1	23.9	33.1	27.5
Age (y)	13.1 ± 2.7	14.5 ± 1.7 *	12.8 ± 2.8	12.7 ± 2.8
Fat Mass (%)	29.8 ± 11.0	36.5 ± 5.1 *	27.1 ± 12.6	29.3 ± 9.7
BMI*z* ^b^	0.9 ± 1.1	1.5 ± 0.3 *	0.9 ± 1.2	0.6 ± 1.1
Fasting Leptin (ng/mL)	15.7 ± 14.9	23.1 ± 9.5 *	13.7 ± 15.3	14.2 ± 15.5

^a^ Loss of control (LOC); ^b^ body mass index *z*-score (BMI*z*); * *p* < 0.01; Note: All data are presented as mean (M) ± standard deviation (SD) unless otherwise noted.

**Table 2:** Loss of control (LOC) Eating, Trait Anxiety, and Interaction Effects on Fasting Serum Leptin.

.	*F*	*β*	*p*-Value	η_p_^2^
Trait Anxiety	3.00	<−0.01	0.08	0.01
LOC ^a^ Eating	5.81	−0.21	0.02 *	0.01
Trait Anxiety × LOC Eating	5.27	0.01	0.02 *	0.01
Age (y)	0.29	<0.01	0.59	<0.01
Race	0.86	0.02	0.36	<0.01
Height (cm)	44.35	−0.01	<0.01 *	0.08
Sex	97.57	0.20	<0.01 *	0.16
Study Type	0.25	0.02	0.62	<0.01
Fat Mass (kg)	1529.06	1.34	<0.01 *	0.75

^a^ Loss of control; * *p* < 0.05.
